# A Lesson of the War

**Published:** 1919-11-08

**Authors:** 


### A LESSON OF THE WAR.

Comparisons are not of necessity odious. They supply the popular judgment with tests which are reliable and easy of application; while they chart out courses for future policy which we may follow without hesitation or misgiving. The years that have passed since the great barriers of peace and goodwill were broken, and the deluge of war descended over the earth, have taught us, not only the strength and fortitude of our souls, but many a good lesson beside. And one of these has been to analyse our own conduct with dispassion, while perceiving virtue in others with neither envy nor rancour. In this spirit we propose to touch upon a comparison between the voluntary hospitals and those which are included in the military system.

We suppose that, never since the world began, has such activity been displayed towards the ills of the flesh, their prevention and cure, as the last five years have shown. And the remarkable feature is that such activity has not been limited to routine, but has comprised a vast advance in mass of all sections of the art of healing. To what has the great awakening been due? May it be attributed to the invasion of the military system by the spirit of civilian medicine, or are the soldier-surgeons and physicians to gain the credit?

We believe, ourselves, that the thing indicates a new birth. Just as fresh and rising civilisations have their origin in the intermixture and blending of two different and perhaps time-worn races, so we regard the new medicine as the offspring of this union, brought about by the stress of war, between the civilian and military therapeutic elements. The champagne of the individualist, the almost irresponsible enthusiasm of unrestrained civil surgery, has infused a freshness and vigour into the Army system which was not there before; while the rigid but efficient machinery of military medicine has provided a sound organisation whereby individual efforts may be directed, controlled, co-ordinated, and, above all, known and perceived by those in authority. There is no necessity to carry it into further detail, or to decide which party shall receive the greater credit. The two parents have equal rights in the child.

And now we come to the end and object of all this argument, which is this, that having beheld, appreciated, and wondered at the outcome of the blend of system with individualism, we cannot but look with favour upon an extension of this union. At present the voluntary hospitals are for the most part without any general organisation at all. Let this want be remedied at once. In the absence of some better co-ordinating system than they now possess, many of the voluntary hospitals, unsustained by the new-born spirit of advance, assuredly will sink into insignificance and disappear. The world will then be the poorer by the loss of their sweet and refreshing influence. Such loss must be prevented. Its prevention is one of the ponderous responsibilities that rest upon the shoulders of the generation of to-day.

